# The Struggle of Neglected Scientific Groups: Ten Years of *NeTropica* Efforts to Promote Research in Tropical Diseases in Central America

**DOI:** 10.1371/journal.pntd.0001055

**Published:** 2011-07-26

**Authors:** Edgardo Moreno, José María Gutiérrez, Esteban Chaves-Olarte

**Affiliations:** 1 Programa de Investigación en Enfermedades Tropicales, Escuela de Medicina Veterinaria, Universidad Nacional, Heredia, Costa Rica; 2 Instituto Clodomiro Picado, Facultad de Microbiología, Universidad de Costa Rica, San José, Costa Rica; 3 Centro de Investigación en Enfermedades Tropicales, Facultad de Microbiología, Universidad de Costa Rica, San José, Costa Rica; George Washington University, United States of America

The general strategy used by high-income countries to address global health challenges in low- and middle-income countries (LMICs) relies heavily on short-term strategies designed to diminish the burden of diseases afflicting the populations of those countries. Thanks to the support and funding of international agencies, in many cases these initiatives have resulted in health improvements. However, in order to have a sustainable impact on the public health of LMICs, “vibrant local scientific communities” need to be implemented in parallel efforts [1]. In this article we describe 10 years of activities of the Network for Research and Training in Tropical Diseases in Central America (*NeTropica*), aimed to develop a competitive Central American scientific community in the field of tropical diseases, with the assistance of the Swedish International Development Cooperation Agency (SIDA) and the participation of the public universities of Central America (CA).

## Historical Background: Central America in the 1980s

Although the official history of *NeTropica* started 10 years ago [2], its roots are tracked to the late 1980s when the Central American universities and the Karolinska Institute bilateral graduate program was initiated [3]. Those were difficult times for Central America. The crisis of the pattern of economic growth that sustained these countries during the post-war period (1945–1979) occurred in the context of a growing political upheaval against the authoritarian regimes of several countries in the region. Between 1950 and the 1970s, the development of Central America was characterized by an economic growth associated with a highly unequal distribution of wealth, with a small high-income class and widespread poverty in the rest of the society [4]. The political context was characterized by military regimes in most of the countries associated with a growing repression of social movements and of political forces that proposed peaceful democratic processes to elect governments. A notorious exception to this pattern was Costa Rica, where a democratic political regime had consolidated for various decades in the context of ambitious social programs that improved living conditions [5]. Panama showed a somehow different and mixed trend when compared to the rest of CA: a progressive and nationalist military regime began in the late 1960s, which improved social conditions and negotiated in 1977 the Torrijos-Carter Treaties aimed at establishing the sovereignty of Panamanians over the Panama Canal by the year 2000 [6].

A different historic, economic, and political cycle started in 1979. In Nicaragua, a mass movement toppled the dictatorship of the Somoza family and paved the way for the Sandinista Revolution (1979–1990) and the onset of the military anti-sandinista movement (the Contras during 1981–1989), which generated a prolonged armed conflict in the 1980s. Likewise, a bloody civil war started in 1981 in El Salvador, lasting for a decade and resulting in more than 70,000 deaths, until the 1992 peace agreement between the Frente Farabundo Martí para la Liberación Nacional and the government. In Guatemala, the armed conflict between the guerrilla movement and the national army escalated during the first half of the 1980s, the repressive nature of the state was reinforced, and there were permanent violations of human rights, particularly in impoverished rural areas. One of the most dramatic consequences of this conflict was the migration of more than 500,000 people, mainly to southern Mexico. Peace agreements were eventually signed in the context of democratic reforms. In Honduras, although democracy and elections returned after 1980, the political influence of the army remained high during those years. Costa Rica and Panama, despite not being directly involved in armed conflicts, were indirectly affected by the regional turmoil as well [7]. On top of this complex political scenario, the region suffered the consequences of the international economic crisis of the late 1970s and early 1980s.

## The Emergence of a Bilateral Graduate Program

The intensification of armed conflicts during the first half of the 1980s and the difficulties associated with the peace negotiations in the region motivated the interest of European countries to contribute to the development of CA. European cooperation intensified after 1984 [8], especially as a consequence of the regional peace agreement signed in Esquipulas (Guatemala) by the presidents of Guatemala, El Salvador, Honduras, Nicaragua, and Costa Rica in August 1987. In this agreement, the basic principles and procedures to solve social and political conflicts in these countries were established after intense negotiations within the region.

One nation that has provided sustained support to the reconciliation and reconstruction of CA is Sweden. In the context of a multifaceted cooperation, two Swedish institutions pursued a novel academic project with CA in medical microbiology: the Karolinska Institute and the Swedish International Development Cooperation Agency (SIDA). The counterparts in the region were the Central American public universities: Universidad de Costa Rica, Universidad Nacional, Universidad de Panamá, Universidad Nacional Autónoma de Nicaragua, Universidad de El Salvador, Universidad de San Carlos de Guatemala, and Universidad Nacional Autónoma de Honduras. These are the most active (and sometimes the only) institutions in terms of research activities in the region. In this context, the Karolinska Institute Research and Training Program (KIRT) and the seven Central American universities provided the academic framework, whereas SIDA supplied the funds for initiating the program.

The first step was to establish a training program between the KIRT and the Central American universities (KIRT/CA) in 1988 [3]. A plan was devised to identify a core group of Central American researchers and students in biomedicine, and to create a graduate program based on joint teaching and collaborative research activities performed in both the Central American universities and the Karolinska Institute. Thus, the involvement of a number of Central American researchers at the local universities was a key element from the very beginning of this program. After finishing the courses and projects, the Central American students defended their thesis in Sweden and the degrees were awarded by the Karolinska Institute, applying the same standards used for regular graduate students. This was the foundation of a “sandwich” model that allowed the Central American students to address, from a competitive perspective, scientific problems relevant to the field of tropical diseases affecting their countries.

The KIRT/CA program ended in 1999 with the graduation of 23 PhDs and 26 MScs. From the MSc group, five additional students obtained their PhDs abroad, in different universities. The nature of the sandwich model promoted a close contact of the trainees with their home countries, with a significant part of the experimental work carried out in the Central American universities. These graduates were acquainted with the conditions they had to confront to perform research in universities characterized by harsh economic constraints and other difficulties. This was the purpose of the sandwich model: to perform quality research in difficult circumstances after being trained in two very different environments. This strategy resulted in a high return rate, with over 90% of the former KIRT students still academically active in Central American universities [2], [9].

## A Step Forward: The Birth of *NeTropica*

Notwithstanding, the insertion of KIRT/CA graduates in their countries highlighted a handicap not foreseen in advance: although these Central American scientists had acquired the abilities needed to perform research, they lacked experience in areas such as fundraising, funds management, and establishing international collaborations [2], [9]. Within this context, the proposal of creating *NeTropica* was born at the end of 1998. The broad idea was to open a window of opportunity for Central American scientists to perform research, and to complement the efforts carried out by the Department for Research Cooperation of SIDA (SIDA/SAREC) and the KIRT/CA programs. Within this context a pilot program was initiated in 1999.

The basic idea of *NeTropica* was inspired in the philosophy of the International Foundation for Science (IFS). Nevertheless, there were differences between these two programs. Instead of having a worldwide scope for low-income nations, *NeTropica* focused only on Central American countries, and supported cooperative research rather than individual efforts. In addition, *NeTropica* concentrated on human health problems, a subject not covered by IFS. The long-term goal of *NeTropica* was to teach young Central American investigators on how to request competitive international grants, and to manage research funds in a region with no experience in these subjects [2].

The operational plan called for the submission of joint research proposals to *NeTropica* from at least two partner groups from different Central American countries. Initially, a Swedish counterpart was also requested in order to provide mentorship to these newly formed consortia. This requirement was, however, rapidly eliminated, as the local Central American groups gained experience in preparing grant proposals and performing research. The proposals were subjected to a strict peer-review process by panels of international experts in the field of tropical diseases and under internationally accepted criteria. Thus, the projects were evaluated mostly on the basis of their scientific merit and collaborative frame. After the selection of projects, the funds were transferred to an institution in CA responsible for financial management. Upon conclusion of the projects, the teams had to demonstrate their achievements by standard scientific parameters, such as publications in peer-reviewed journals. After intense negotiations, and a 1-year trial, SIDA approved support for *NeTropica,* and official activities began in 2000.

The hypothesis that has driven *NeTropica* efforts has been that control of maladies can only be achieved if scientific groups settled in their countries investigate and understand the key determinants of diseases affecting their population. To accomplish this, it is necessary to perform high-quality research locally and to establish fruitful international collaborations [1], [10].

## Strengthening of the Central American Scientific Community

**Figure 1:**
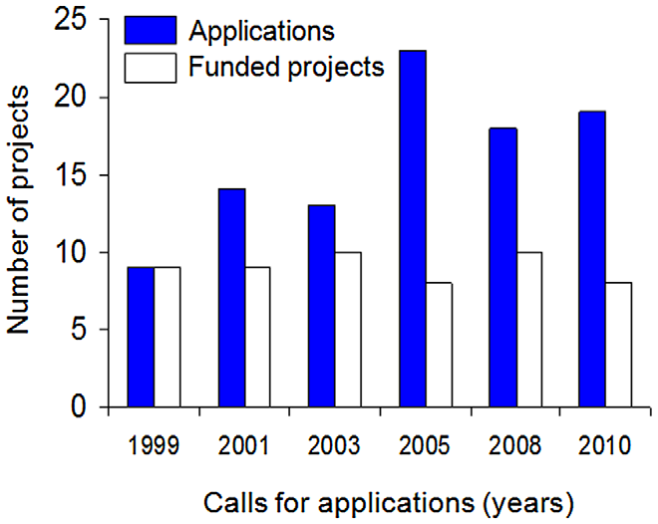
Applications received and grants awarded by *NeTropica* between 1999 and 2010. The average number of scientists per applied/funded project is three.

**Figure 2:**
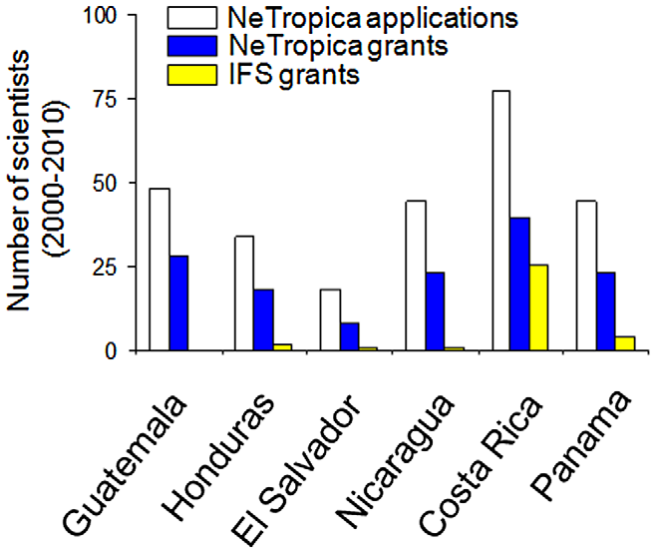
Distribution of grants awarded by *NeTropica* and IFS to scientists of the various Central American countries. The number of IFS awards granted to Central American scientists from 1999 to 2010 countries was taken from the IFS Web page ([14]; http://www.ifs.se/Database/search.asp).

During 10 years of activities, *NeTropica* has run six calls for applications. Relatively few proposals were received in the first rounds, thus resulting in a high success rate for those who applied (Figure 1). Fortunately, there has been a consistent increase in the interest of the Central American scientific community to apply for *NeTropica* funds. Although, this has resulted in a lower successful rate, there has been an overall increase in the quality of the selected projects, as expected (Figure 1). To date, 54 grants (each averaging US$35,000) have been allocated to scientific consortia of Central American teams. There have been differences in the number of applications received from Central American countries, with a high participation of Costa Rican scientists, followed by a robust and homogenous participation from the scientific communities of Honduras, Guatemala, Panamá, and Nicaragua, whereas El Salvador has lagged behind (Figure 2).

**Figure 3:**
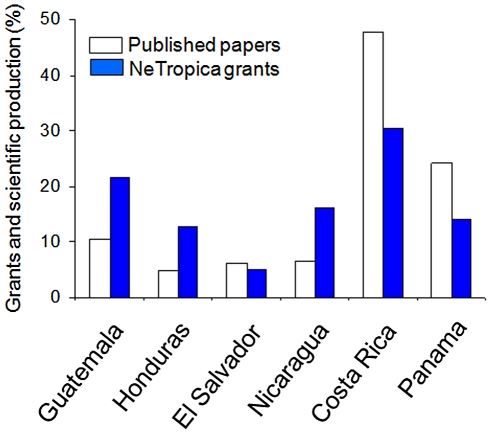
Distribution of *NeTropica* funds to CA countries in relation to their overall scientific productivity. The percentage of the funds allocated by NeTropica from 1999 to 2010 per country is shown. The percentage of the overall scientific production of CA countries from 1996 to 2008 was calculated from data of the SCImago Journal & Country Rank database ([11]; http://www.scimagojr.com/).

The success rate of applications has ranged from 58% (Guatemala) to 44% (El Salvador). There is a significant difference in the number of grants allocated by *NeTropica* from 1999 to 2010, as compared to those allocated by IFS in the same period (Figure 2). This gap is even larger if compared to those grants assigned by the Third World Academy of Sciences (TWAS) (unpublished data). Despite the differences in the scope of research funded, these divergences reflect the profound penetration that *NeTropica* has achieved in the Central American scientific community in this relatively short period of time. Since the funds are allocated based on the scientific merit of the proposal, it is to be expected that those countries having more consolidated scientific communities would be granted a higher percentage of the grants. Indeed, this has been the case, since Costa Rica, Panamá, and Guatemala have received a higher proportion of the funds (Figure 3). However, the difference in funding is not as high as the difference in the relative competitiveness of the countries, measured as the percentage of the total scientific production (Figure 3). This indicates that the strategy followed by *NeTropica* has had the tendency to “democratize” the outcome. This is probably due to the application format, which encourages consortia rather than individual efforts.

**Figure 4:**
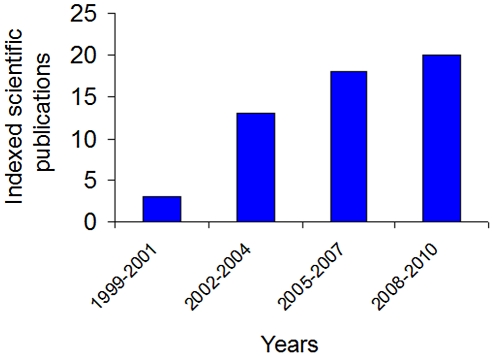
Scientific productivity of *NeTropica* consortia expressed in number of indexed published articles.

In terms of results measured by standard scientific criteria, the sponsored consortia have generated 54 indexed scientific documents that explicitly acknowledge *NeTropica.* There has been an increasing trend in the number of published papers, thus demonstrating that the Central American scientific community is steadily incorporating internationally accepted criteria to measure scientific performance (Figure 4). The articles generated by the consortia supported by *NeTropica* have been published in a variety of well-recognized journals with a mean impact factor of three. A search for scientific production in CA at the SCImago Research Group database from 1996 to 2008 under the terms “microbiology and immunology”, “infectious diseases”, and “medical microbiology” indicated the generation of between 98 and 560 articles [11]. Thus, although the number of papers published with the support of *NeTropica* funding seems a modest outcome from standard international criteria, in the poorest scenario it corresponds to 10% of the published scientific papers in these disciplines in CA.

In addition to the core activity based on research grants, *NeTropica* has also promoted regional graduate programs in Costa Rica and Honduras, with ten fellowships awarded to Central American students to enroll in local MSc and PhD programs. It is expected that these graduate programs will decrease the regional dependency on foreign graduate programs for the training of local students. At the same time, the students enrolled in these programs would contribute to the local generation of science on health subjects relevant for the region [12].

The aims of *NeTropica* are complemented by activities that promote the academic atmosphere in the region, which are essential to generate an active scientific community. Among those, *NeTropica* has organized six international scientific meetings and two workshops on fundraising and intellectual property issues. Furthermore, it has contributed to the development of an efficient system for the management of research funds in CA. Several of the groups sponsored by *NeTropica* have become leaders in biomedical research in their countries. In many cases, they are the strongest Central American teams studying specific life-threatening tropical diseases and, in some instances, the only ones. *NeTropica* has also established synergies with various scientific groups outside the region to obtain funds for research activities. Within this international cooperative scenario, *NeTropica* has received the collaboration of many high-level scientists who evaluate proposals, and contribute with ideas and partnerships.

The advantage of involving resident teams in the study of tropical diseases lays in the hypothesis that they, more than others, know the social, economic, institutional, and political contexts in which these diseases occur. Consequently, they should be able to study these pathologies from an integrative perspective, thus helping to envision the strategies more likely to succeed for controlling health problems. It is unlikely that complex maladies such as dengue, trypanosomiasis, various zoonoses, or water-borne diseases, among others, could be understood with *avant-garde* research performed in rich countries alone [10]. Rather, they would need the concourse of high-quality integrative research performed globally, with the involvement of low-income countries. The model of *NeTropica* is based on the premise that excellence in scientific research is fundamental for development. Although this is an obvious internationallyaccepted concept for high-income countries, it has been difficult to put forward in CA owing to chronic weaknesses in regional science development policies.

In course, strong local research groups can develop resident-based scientific knowledge and interact through innovative partnership modalities with laboratories in low- and high-income countries. Clearly, nations capable of performing preventive measures in public health are those that have a pool of investigators in different areas able to develop and adapt new diagnostic, preventive, and therapeutic tools to resolve local problems [1], [10].

## The Need to Consolidate a Successful Regional Project

*NeTropica* is a small agency that deals only with few of the problems faced in CA. The main difficulty is that most of the groups in CA do not have access to research funds, which are scarce or non-existent in most of these countries. The availability of international resources for Central American scientists is limited: on the one hand, Central American groups are not yet strong enough to apply for competitive international funds and, on the other hand, the global agencies usually limit these groups to small funds, focused on “applied”, translational, or immediate problem-solving research. While the former issue is a “redundant endless loop”, the latter does not allow local researchers to address primary scientific questions [13]. Moreover, a large proportion of funds provided by international agencies to investigate neglected diseases prevalent in low-income countries have been directed to large research groups in high-income countries [10]. The assumption that these diseases can be understood without the involvement of local groups in low-income countries is misleading. Within this context, the long-term vision of SIDA has made a significant impact in CA through its bet in supporting an agency that allocates research funds to local groups based on scientific merits [2], [3].

As with other agencies, *NeTropica* depends on economic resources to pursue its goals; however, it belongs to a region characterized by a shattered economy. Moreover, in spite of the fact that most of the original 1980s hostilities have been settled, social inequity and political conflicts persist in the area, indicating that CA still struggles for political and economic stability, as well as for social development. These are major obstacles that constrain *NeTropica*'s sustainability. *NeTropica* works locally, in a neglected geographical area, within the agenda of neglected diseases investigated by neglected research groups. In 10 years, *NeTropica* has demonstrated commitment, efficiency, and good results in promoting scientific research in CA. This scientific capacity has contributed to a better understanding of relevant diseases in this region. The main goal of *NeTropica* has not just been to move towards the prevention and cure of life-threatening diseases in the region but, above all, to build scientific capacity and to support groups that could perform these duties. Taking into consideration the significant achievements of *NeTropica* during its short life span, the strengthening of this regional effort, with the participation of local and international partners, will undoubtedly contribute to the improvement of public health and science in CA.
